# New perspectives for viability studies with high-content analysis Raman spectroscopy (HCA-RS)

**DOI:** 10.1038/s41598-019-48895-7

**Published:** 2019-09-02

**Authors:** Abdullah Saif Mondol, Natalie Töpfer, Jan Rüger, Ute Neugebauer, Jürgen Popp, Iwan W. Schie

**Affiliations:** 1 Leibniz Institute of Photonic Technology and Leibniz Health Technologies, Albert Einstein Str. 9, 07745 Jena, Germany; 2 Institute of Physical Chemistry and Abbe Center of Photonics, Friedrich-Schiller University Jena, Helmholtzweg 4, 07743 Jena, Germany; 3 Center of Sepsis Control and Care, Jena University Hospital, Am Klinikum 1, 07747 Jena, Germany

**Keywords:** Biochemical assays, Lab-on-a-chip, Raman spectroscopy

## Abstract

Raman spectroscopy has been widely used in clinical and molecular biological studies, providing high chemical specificity without the necessity of labels and with little-to-no sample preparation. However, currently performed Raman-based studies of eukaryotic cells are still very laborious and time-consuming, resulting in a low number of sampled cells and questionable statistical validations. Furthermore, the approach requires a trained specialist to perform and analyze the experiments, rendering the method less attractive for most laboratories. In this work, we present a new high-content analysis Raman spectroscopy (HCA-RS) platform that overcomes the current challenges of conventional Raman spectroscopy implementations. HCA-RS allows sampling of a large number of cells under different physiological conditions without any user interaction. The performance of the approach is successfully demonstrated by the development of a Raman-based cell viability assay, i.e., the effect of doxorubicin concentration on monocytic THP-1 cells. A statistical model, principal component analysis combined with support vector machine (PCA-SVM), was found to successfully predict the percentage of viable cells in a mixed population and is in good agreement to results obtained by a standard cell viability assay. This study demonstrates the potential of Raman spectroscopy as a standard high-throughput tool for clinical and biological applications.

## Introduction

Raman spectroscopy has attracted a lot of interest as a versatile tool for clinical and biological applications due to its ability to provide molecular fingerprint information of tissue and cells in a label-free manner^1–3^. The method is based on an inelastic light scattering event between a photon and a molecule, which results in a wavelength change, accurately describing the chemical composition of a sample. Furthermore, due to its label-free nature it requires little to no additional sample preparation and can be used to study living cells. Several applications, e.g. investigation of drug cell interaction^4–6^, determination of cell stages^7^, cell death due to toxicity^8^, diagnostics^9^, biomedicine^10^ and other clinical applications^11^, have already been demonstrated, showing exceptional accuracy and novel insight into molecular changes in these model systems. Nevertheless, obtaining acceptability of Raman spectroscopy as a standard bio-laboratory tool has been rather a slow process, largely due to prolonged measurement times, low number of measured samples, complex data analysis procedures, and the vast human effort, which is required to perform the experiments. Current state-of-the-art Raman systems have a high data acquisition rate, i.e. read-out-rate, which is specifically related to the read-out speed of the detector^12^. However, the measurement covers an entire process of sampling large number of cells, which involves determination of the correct focal plane, identification of the randomly distributed cell positions on the substrate, translation of the cells in the laser focus, acquisition of a spectrum from the cell, saving the spectral data, and repeating this process for all cells of interest. In a recent publication, we have developed a high-throughput screening Raman spectroscopy (HTS-RS) platform that allowed the acquisition of average Raman spectra of cells in an automated fashion, removing user interaction during the data acquisition procedure, resulting in an extreme boost in throughput and rapid sampling of thousands of cells in a short period of time^13^. The HTS-RS approach truly harvests the advantage of Raman spectroscopy; because minimal sample preparation is required and large, statistically significant sample sizes of a mixed population sample can be reached quickly. In comparison, conventional investigation of single cells using Raman spectroscopy only measured a few hundred cells, and rarely in mixed populations, significantly limiting a translation to real applications. The overall cell information or the average Raman spectra of the cells are acquired by illuminating the cell with a laser spot size similar to the dimension of the cell – hence the necessity of cell imaging is omitted sparing time and effort. Nevertheless, the HTS-RS platform is restricted to perform one experiment at a time without user interaction. However, a large number of applications require the characterization of samples under different physiological conditions, i.e. performing a series of experiments. Typically, these kinds of tests are performed in multiple well plates. For example, the evaluation of chemotherapeutic drug efficacy is a major step in cancer patient treatment and the personalized medicine. In the testing of chemotherapeutic drugs, such as the well-established doxorubicin (DOX)^14^, THP-1 cells, a monocytic acute myeloid leukemia cell line, have been previously used as a model system^15^. Various cells have been shown to be susceptible to the effect of DOX, e.g. breast cancer^16^, lung cancer^5^, leukemia cells^6^.

Although Raman spectroscopy has been widely used to investigate drug cell interaction^4–6,17–21^, as of now, the experimental procedure has been cumbersome and with several draw-backs. The cells have to be measured by a trained user either in mapping mode or from random locations within the cells^7,17,19,22,23^. Raman spectroscopy has advantages compared to traditional methods, admittedly, the human factor here not only increases the probability of measurement error, but also limits acquiring substantial amount of data for proper statistical evaluation. The previously presented HTS-RS can overcome these disadvantages of the current Raman-implementation and can provide a significant benefit, because it removes the human dependency from the data acquisition and a substantial amount of data can be accumulated for an explicit statistical evaluation. In this work, we have extended the previously proposed HTS-RS platform to allow the measurement of cells under different physiological conditions, and used the high-content analysis Raman spectroscopy (HCA-RS) platform to investigate drug-induced changes in cells. This new extension – HCA-RS - opens a new way to rapidly assess the molecular signature of cells exposed to different physiological conditions. To demonstrate the advantages of the proposed approach, a Raman-based drug-assay validating the effects of doxorubicin (DOX) concentrations on THP-1 cells was implemented. Based on the acquired Raman spectra and proper statistical modelling, we were able to analyze the effect of DOX on the cells as well as predict the cell viability ratio in a mixed population. Here, the mixed population is a sample containing both viable and non-viable cells. Our findings using the HCA-RS platform were validated against a standard clinical viability test, showing exceptional reproducibility with the developed approach. The newly proposed HCA-RS platform creates a paradigm change for the application of Raman spectroscopy for the molecular analysis and characterization of eukaryotic cells.

## Results

### Development of HCA-RS platform

The HCA-RS platform requires minimum user input, i.e. the laser power, integration time, number of image frames per well, and number of wells corresponding to different physiological conditions. This simple set of parameters is sufficient to perform the experiments and also bears an enormous potential for standardization of experimental procedures. In this work, the parameters were 96 mW laser power (λ = 532 nm) in the sample plane, 0.25 seconds of integration time, 25 frames for each well corresponding to an area of 800 × 600 µm^2^, and six wells, containing either cells incubated with different DOX concentrations or control cells. The measurement process is a combination of iterative steps, incorporating acquisition of a bright-field image, and the computational localization of cells in the image, followed by automated translation of each cell into the laser focus, data acquisition, and saving of the data^13^. The HCA-RS approach integrates measurement of further wells upon completion of the spectral acquisition from a single well in a consecutive way. If additional wells containing further samples exist, the translational stage moves the next well below the objective and measurements are performed with identical parameters. The whole process continues until all samples under different physiological conditions have been measured. The distance between the neighboring wells are 10.5 mm and 14.1 mm along the x and y direction, respectively. This corresponds to the dimension of the CaF_2_ cover slip (Crystal GmbH, Germany), which was used as the sample carrier for the measurements. In each sample well an in-house written auto-focus algorithm first determines the z position of the best focal plane, and is repeated at a different location of the same well to compensate for tentative tilting of the cover slip. For each frame, a bright-field image is captured and the positions of the existing cells are recorded by using an in-house developed cell detection algorithm^13^. When spectra of all cells in one bright-field image or a field of view (FOV) are obtained, the stage translates to the next FOV and the same routine is repeated.

Besides the integration time for the Raman measurement, user has an option to select waiting time before the data acquisition. This can be of a great advantage in applications where the samples exhibit autofluorescence and require additional bleaching for significant signal-to-noise ratio improvement. Here, the DOX treated cells showed large fluorescence leading eventually to saturation of the spectrometer CCD camera as the absorption of DOX at 532 nm is high^24^. In the present study a waiting time of 5 s was selected. At low laser power the reduction in fluorescence level after photobleaching was not sufficient to acquire a qualitatively good Raman signal. Especially the Raman signal from cells incubated with higher DOX concentrations mostly saturated the CCD camera. Therefore, a combination of elevated laser power, short integration time, and additional photobleaching time enabled acquisition of qualitatively acceptable Raman spectra.

With the HCA-RS platform two biological replicates of DOX treated THP-1 cells were investigated. In total 25031 cells were sampled and a comprehensive overview of the whole data set is given in Table 1. The number of observable cells in the bright-field images decreases with increasing DOX concentration, which was already indicating a cytotoxic response. Two different sets of control samples were measured in the beginning and at the end of each run, to estimate the effect of possible time and device-related shifts in the Raman spectra. No such shifts were observed in spectra from control samples throughout the entire acquisition procedure. After incubation of DOX treated cells, a standard viability test was performed – details are presented in the Methods section. At the time point of cell fixation, the cells were viable and non-viable. In remaining of the manuscript, the cells are labeled accordingly.

**Table 1:** Number of investigated cells per sample for two biological replicates using HCA-RS system.

Drug concentrations	Batch 1 Number of cells	Batch 2 Number of cells	Total Number of cells
Control (0 µM)	2560	2951	5511
0.05 µM	2482	2911	5393
0.1 µM	1934	1908	3842
0.5 µM	1035	1313	2348
1 µM	914	1358	2272
Control (0 µM)	2568	3097	5665
**Total**	**11493**	**13538**	**25031**

The two controls are untreated THP-1 cells, and the other samples are THP-1 cells exposed to different concentrations of DOX for 48 hours. The concentration levels of DOX are indicated in the left column. The automated measurement order was from top to the bottom.

### DOX induces raman-measurable changes in cells

**Figure 1:**
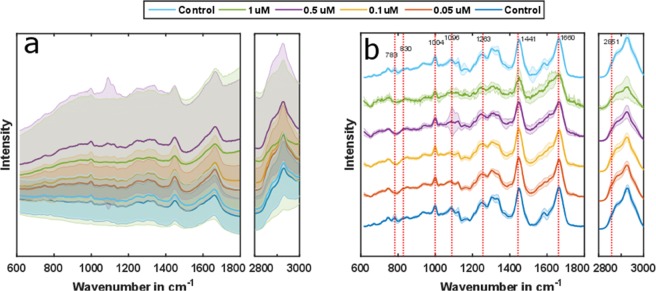
The mean Raman spectra and corresponding standard deviations of samples for a chosen spectral range are shown before (**a**) and after the background correction (**b**). Although there is some spectral information detectable before background correction, more spectral features are evident in the background corrected spectra. Some changes in the spectral bands for different DOX concentrations are visually observed, indicating intracellular macromolecular variations.

To estimate the DOX-induced changes in cells, the acquired Raman spectra were preprocessed, based on the strategy outlined in the Methods section. After the preprocessing, the spectral regions of interest, i.e. 615 cm^−1^ to 1800 cm^−1^ for the fingerprint region, and 2790 cm^−1^ to 3010 cm^−1^ for the high wavenumber region, were employed for further analysis, while the silent region was omitted. As DOX has strong absorption-emission properties around 532 nm^25^, internalization of DOX in the cells would lead to pronounced fluorescence with increasing DOX concentration. This was evident in the acquired signal (Fig. 1a) from the cells. The fluorescence contribution was removed, using spectral preprocessing (Fig. 1b). Additionally, spectra not containing information due to erroneous cell detection and occurring at a frequency approximately one out of ten, were excluded from the dataset before further analysis. The Raman spectra from two background corrected control samples show no significant differences, confirming no major influence from the time lapse in between the measurements (Fig. 1b). Changes in several vibrational bands are evident in the background corrected spectra of DOX-treated cells and can be related to compositional alterations in the macromolecular pool, i.e. protein, lipid, nucleic acid, and DOX.

Bands associated with the major macromolecules, such as nucleic acids, proteins, and lipids can be found in the cell spectra. Typical nucleic acid bands are present at 783 cm^−1^ (uracil, cytosine, thymine ring breathing), 813 cm^−1^ (RNA O-P-O stretching), 830 cm^−1^ (DNA B-form O-P-O asymmetric stretching), 1096 cm^−1^ (DNA PO_2_ symmetric stretching)^5,26^. Protein related bands are found at 760 cm^−1^ (tryptophan), and signatures of tyrosine ring-breathing mode are found at 830 cm^−1^ ^26^. Furthermore, the symmetric ring breathing mode of phenylalanine, a typical protein marker band, can be found at 1004 cm^−1^. Amide I and amide III can be found at 1660 cm^−1^ and 1263 cm^−1^, respectively^5,6,26^. Here, 1263 cm^−1^ (=C-H in-plane cis) and 1660 cm^−1^ (C=C cis double bond stretching) are also related to lipids^27^. Further lipid signatures are found at 1096 cm^−1^ (chain C-C stretching), at 1303 cm^−1^ (C-H_2_ twist)^26^, 1441 cm^−1^ (C-H scissor) and 2851 cm^−1^ (CH_2_ symmetric stretching)^27^. The 2851 cm^−1^ band is commonly used in coherent anti-Stokes Raman spectroscopy for lipid droplet analysis^28^. In^5^, the doxorubicin bands are assigned to 1247 cm^−1^ (C-H bond of DOX), 1215 cm^−1^ (C-O-H bond of DOX) and 1086 cm^−1^ (C-O bond of DOX). Higher DOX concentrations correlate spectral changes at 783 cm^−1^, 830 cm^−1^, 1004 cm^−1^ and 1247 cm^−1^, which can be mainly assigned to nucleic acid, protein and DOX content. Farhane *et al*. readily reported physiological effects of DOX on nucleic acid changes in lung cancer cells, such as DNA denaturation, cessation of DNA replication, and DNA intercalation of the drug^19^. Further changes in protein bands can be due to changes related to apoptosis^16,19^. The difference in lipid content can be monitored by the Raman band at 2851 cm^−1^ and indicates pronounced formation of lipid vesicles at the cell surface as well as blebbing of the cell membrane, a known process in apoptosis^8,18^.

**Figure 2:**
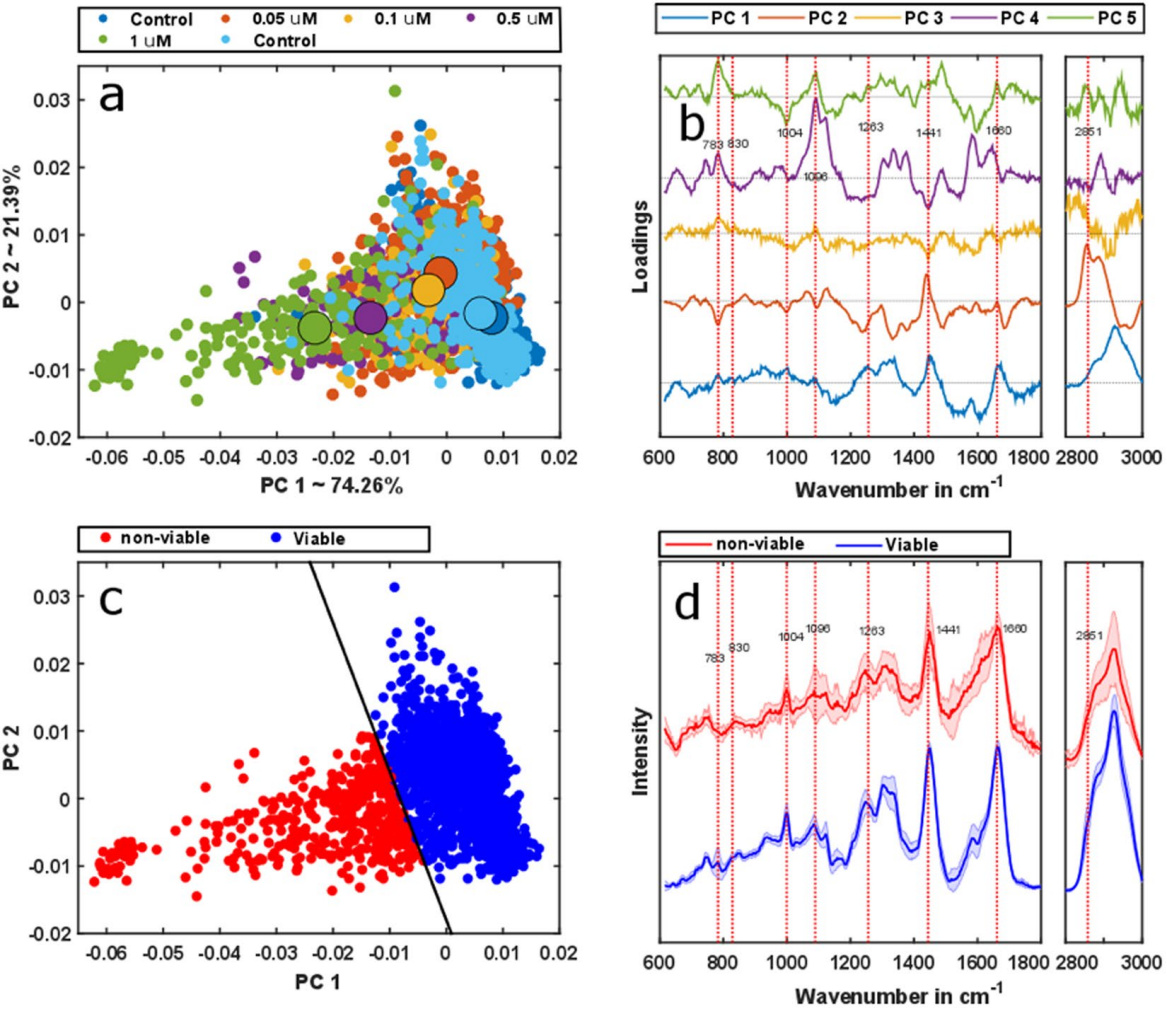
PCA was applied to the background corrected spectra. The scores plot of the first two PCs indicates the changes corresponding to the DOX treatment (**a**). The corresponding described variances by PC1 and PC2 are mentioned in the axis label (**a**). (**b**) Loadings of the PCA model. The scores corresponding to the first two loadings, PC1 and PC2, were employed for the SVM model. Afterwards, the SVM model was applied to the dataset. (**c**) The prediction of PCA-SVM separates viable and non-viable cells. The color-coding is done based on the PCA-SVM model prediction whereas the model regions are separated by the decision boundary – solid black line. (**d**) The mean spectra of corresponding predicted classes by the SVM model also show pronounced spectral differences in several band positions.

The effect of DOX on the cells can be analyzed more effectively, using principal component analysis (PCA). PCA is a useful technique to reduce the dimensionality of the dataset^29^, where new orthogonal components are ordered based on their corresponding variances, i.e. the first several principal components account for most of the variance observed in the dataset. The method has been extensively used to analyze Raman spectra^19,30,31^, as it helps to determine even minimal spectral variations. The score plot of the first two principal components reveals a clear transition regarding the medians of corresponding point clouds from spectra of control samples and those containing higher DOX concentrations (Fig. 2a). As can be seen in the scatter plot, the scores of the two controls (blue and cyan points) overlap, forming clusters on the positive principal component 1 (PC1) axis, thus, any time-dependent, device-related influence during the measurement can be neglected. Medians of point clouds from samples with higher DOX doses are prominently shifted towards negative values of PC1. The first five loadings are plotted in Fig. 2b, and provide insight into macromolecular and intracellular changes induced by DOX. The loading of PC1 resembles a combination of the protein and the nucleic acid spectra^32^. These two macromolecular components are mainly responsible for the changes in score plot along PC1. On the other hand, the second loading can be seen as a combination of the lipid and the nucleic acid spectra^32^. They induce the changes along the PC2 axis. Interestingly, the spread along PC1 is more dominant than along PC2. The score plot already implies a striking interrelation of spectrally verifiable alterations in the macromolecular composition of cells upon higher DOX concentrations exposure. The cell viability is also influenced by the DOX exposure, which is also evident in Raman spectral signature – addressed in the following.

### Differentiation of viable cells

DOX is also widely employed to induce apoptosis – hence reduces the cell viability^14,16–19^. Although, Raman spectroscopy can differentiate various cell death mechanisms, i.e. apoptosis or necrosis^26,33^, in the present study only the cell viability was tested upon the exposure to DOX. Raman spectroscopic detectable changes in cellular makeup, as outlined in the previous section, are a direct consequence of DOX-induced cell viability alteration in THP-1 cells and can, therefore, be conveniently studied by this modality. Although PCA significantly reduces the dimensionality of the dataset and renders a better visualization method for the spectral change compared to the mean spectra, this unsupervised method is challenging to use for classification problems. To address this issue, i.e. to differentiate between viable and non-viable cells, supervised machine learning algorithms, support vector machines (SVM) were combined with PCA. SVM separate objects of distinct classes in a dataset using an optimal decision boundary, a hyperplane^34^. As the optimal hyperplane is obtained by maximizing the margin in between the classes with the support vectors, SVM provide a robust classification model that is less sensitive to outliers. Several studies have demonstrated the applicability of SVM for Raman spectral data analysis^33,35–37^. Clinical diagnostics of leukemia cells from healthy volunteer as well as patients are also investigated using SVM^38^. In this work first, the SVM classifier was trained with two separate classes, i.e. viable and the non-viable cells using a linear classifier with an alpha value of 0.5 and a solver that follows the iterative single data algorithm^39^. This model was applied to a test dataset and their classes are predicted. The first control – the untreated THP-1 cells and the cells with 1 µM DOX treatment from batch 2 (biological replicate 2) were taken as the training dataset, as their cell viability ratios were 99% and 13%, respectively (Table 2). Because all of the presented measurements here were performed on a mixed population, it was paramount to perform clinical validation of the achieved results. Corresponding values were estimated by the standard clinical cell viability test, which is outlined in the Methods section. The first two principal components were used to build the SVM model, which constitute 95.65% of the total spectral variance in the dataset. The first and the second PCs correspond to 74.26% and 21.39% of the spectral variance, respectively. The model cost also known as the estimated error, was obtained by a ten-fold cross validation process, and estimated to be 4.89%. The SVM model was then applied to Raman spectra from both of the biological replicates, 1 and 2, predicting the cell viability states as well as their percentage contributions in the population. Afterwards, it was compared with the clinical cell viability tests (Table 2), where the deviations in between the two approaches lie within the reliability limit of a clinical viability tests^40^. The corresponding IC_50_ value of the Raman data from both batches is 0.199 which is close to values reported in the literature for other DOX-treated cells^4,5^. The deviation of the IC_50_ value to literature values may be due to limited data points, as evident in the viability curve presented in Fig. S2 in supplementary document. The score plot for SVM prediction shows a good separation between the viable and the non-viable cells where the decision boundary is indicated by a solid line (Fig. 2c). The corresponding mean spectra of the two distinct classes also demonstrate spectral features (Fig. 2d), e.g. changes at 783 cm^−1^, 1004 cm^−1^, 1740 cm^−1^, which can be related to changes in the macromolecular content. Up to this point, the SVM model separates the viable cells and non-viable cells. The effect of DOX concentration to individual type namely viable and nonviable cells can also be estimated using the Raman spectra, as elaborated in the next section.

**Table 2:** Values of predicted viability percentages of THP-1 cells treated with different DOX concentrations in comparison with data from independent cell viability tests.

Drug concentrations	Batch 1		Batch 2	
	Raman Results in %	Viability test in %	Raman Results in %	Viability test in %
Control	85	82	99	99
0.05 µM	81	78	78	70
0.1 µM	73	69	69	59
0.5 µM	64	59	36	32
1.0 µM	63	47	22	13
Control	91	85	97	95

### Cell viability-drug concentration dependence

**Figure 3:**
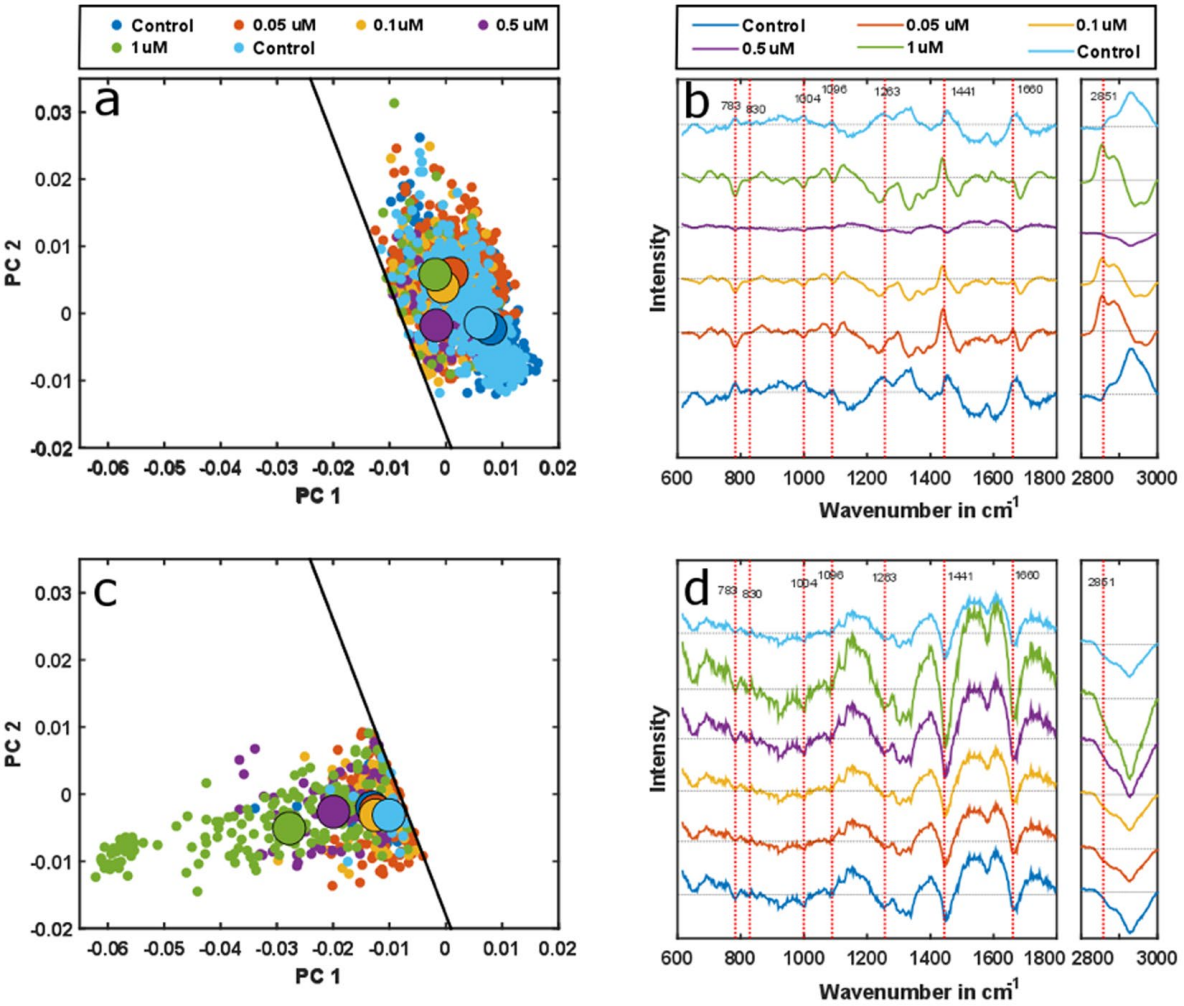
The effect of increased DOX concentration on the viable THP-1 cells (**a**) shows a consistent shift from control cells. The shift is more dominated along PC2, which is related to changes in lipid and nucleic acid content and is supported by the reconstructed spectra (**b**). DOX also influences the non-viable cells, with the influence being more significant at high DOX concentrations (**c**). The scores transit along PC1, which is related to nucleic acid and protein content. The reconstructed spectra also show variations in several band positions (**d**) accordingly. The solid black lines in (**a**,**c**) are the decision boundary in between the viable and non-viable cells obtained from the PCA-SVM model.

Cell viability is reduced with the increase of DOX treatment concentration (Table 2). It induces cellular macromolecular changes both for viable and non-viable cells (Fig. 3a,c). The SVM model readily separates both of the cell types, using the first two PCs (Fig. 2c). The spectra, which correspond to each point in that score plot, were reconstructed by multiplying the two scores of each cell with the corresponding first two loadings to investigate further the underlying spectral characteristics for the separation. The mean value of the spectral dataset was not added to the reconstructed spectra as the target was to find the reason behind the spectral variation. Also, the two groups – viable and nonviable cells, as classified by the SVM model - are sub-grouped depending on their DOX treatment concentration.

The median points in the scores plot of the viable cells shift towards the decision boundary, i.e. the cells are close to become non-viable with increasing DOX concentration (Fig. 3a). The shift occurs due to the spectral changes in various bands, which is evident from the reconstructed spectra (Fig. 3b). Interestingly, all spectra of DOX-treated cells reveal similar patterns of maxima at distinct band positions that correspond to minima in spectra of control cells, e.g. 2851 cm^−1^ and vice versa, e.g. 783 cm^−1^, 1004 cm^−1^. The 1 µM DOX-treated cells deviate from this trend, which may be explained by the low number of viable cells in the cell population. The transition of the median value of scores for increased DOX concentration in the viable cell population is dominant along the PC2 axis and related to compositional changes in the lipid and the nucleic acid contents, which is evident from the second PCA loading (Fig. 2b). This is also in agreement with the apparent accumulation of lipids and the decrease of the nucleic acid features for increasing DOX exposure in Fig. 3b. For the non-viable cells, the highest concentration of DOX, i.e. 1 µM, shifts the median point in the scores plot away from the decision boundary while the low concentration median values remain close to one another (Fig. 3c). The transition of the scores is dominated along PC1, which is related to the change of protein and nucleic acid – evident from the first PCA loading (Fig. 2b). The reconstructed spectra confirm this by demonstrating changes in several band positions concerning the protein and nucleic acid content (Fig. 3d). In contrast to reconstructed spectra of viable cells, spectra of non-viable ones display the same pattern that scales up with the applied DOX concentration, i.e. the absolute spectral changes increase with higher DOX doses.

**Figure 4:**
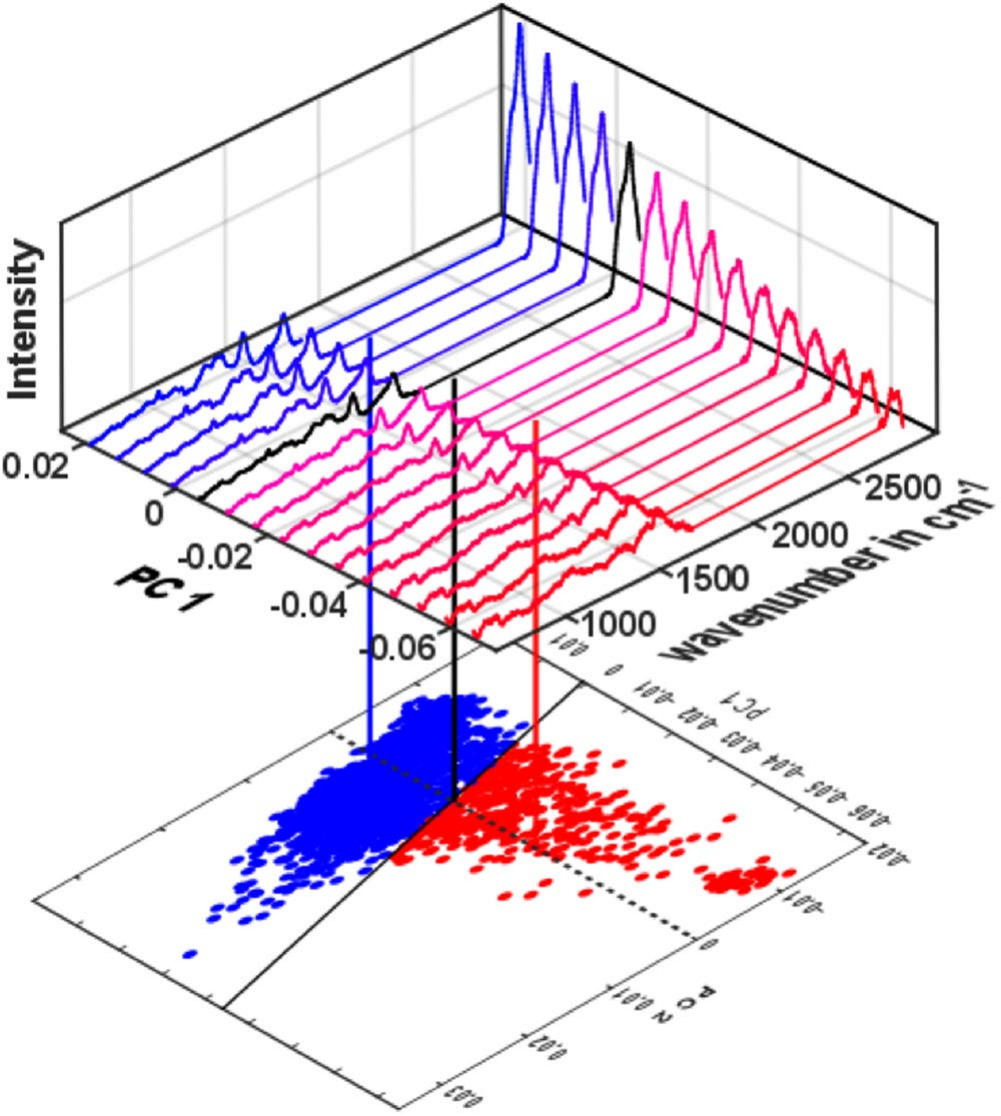
Color-coding of the score plot is done based on the PCA-SVM model prediction, where the corresponding model regions are separated by the decision boundary (solid black line). The scores along the dashed black line, parallel to the PC1 axis in the score plot are used to compute the corresponding spectra. The transition along this dashed line from non-viable to the viable side (from red towards blue region) illustrates the changes in the Raman bands for the two viability states. The black spectrum whose scores lay on the decision boundary represents a threshold. Thus, the cell viability can be related to Raman bands of cellular macromolecules content.

The PCA-SVM model not only differentiates the viable and the non-viable cells in the mixed population, but also helps to evaluate the DOX concentration effect on these two classes individually. For viable cells, the increased uptake of DOX shifts Raman spectra of cells closer to the decision boundary. For non-viable cells, on the other hand, the increase of DOX concentration shifts the cells beyond the decision boundary. This implies non-viable cells, nevertheless, experience macromolecular changes with elevated DOX concentration. To see where the decision boundary lies in the spectral domain, spectra, whose scores fall on a projected line parallel to the PC1 axis in the score plot (Fig. 4), are computed, reconstructed and plotted. The spectra shown in Fig. 4 are similar to the reconstructed spectra in Fig. 3b,d in every aspect; only the mean value is added to the reconstruction process. This visualization allows a more intuitive interpretation of spectral changes due to DOX exposure and emphasizes changes in the macromolecular content of the cells, which ultimately determines whether the cell belongs to the viable or non-viable category. This also demonstrates that the PCA-SVM classification based on acquired Raman spectra not only enables the differentiation of cell types in mixed populations, but also sheds light on the corresponding macromolecular changes.

## Discussion

The total number of acquired spectra (Table 1) demonstrates that the HCA-RS platform can sample substantial numbers of cells with minimum human interaction, providing an in-depth molecular profiling of individual cells at different physiological stages and in a label-free manner. The platform can perform a series of measurements independently, significantly outperforming traditional Raman-based platforms for cell analysis. The total number of measured cells of both biological replicates was more than 25.000 (Table 1). Generally, Raman measurements use long exposure times, apply mapping techniques and later use average spectra for further analysis – hence, resulting in overall long acquisition times, time-consuming manual measurement procedures, as well as a small sample size, e.g. in^20^ from each batch of MCF-7 cells treated with Docetaxel, 2–6 cells were imaged with an integration time of 10 s. Only the acquisition time for the spectra was more than two days. In HCA-RS, the same information can be obtained within less than a minute. The expanded laser spot size in the sample plane of the HCA-RS enables collection of the average spectral information directly eliminating the time consuming Raman mapping process. Furthermore, the automation allows probing more cells in a sample and hence, increases the statistical stability, reduces the human factor to minimize the error and above all makes it more suitable for clinical operator to use the device without any prior knowledge of Raman spectroscopy.

Raman spectra of the two controls (untreated cells) show reproducible spectral signatures (Figs 1b and 2a), indicating that the time-lapse between the measurements does not have major influence on the spectral characteristics. Raman spectroscopic results were validated with a conventional cell viability-assay, showing highly comparable results. The total numbers of cells decreased with increasing DOX concentration (Table 1). This finding can be readily explained as DOX intercalates the DNA and prohibits cells from proliferation^41^, resulting in a lower number of cells present in the sample after 48 hours of treatment. The effect of different DOX concentrations on cells is also deducible from the Raman spectra. A reduction of nucleic acid related bands is observed in both, the background corrected spectra (Fig. 1b) and the reconstructed spectra (Fig. 3b). This observation is in agreement with the proposed DOX-cell interaction mechanisms^41^ leading to DNA denaturation, cessation of DNA replication and DNA intercalation, readily reported in previous studies^6,19^. In^6^, with the exposure of 1 µM DOX for 48 hours to the Jurkat T cells, a strong decrease of nucleic acid (783 cm^−1^, 1096 cm^−1^) as well as change in protein (1004 cm^−1^, 1263 cm^−1^, 1660 cm^−1^) and lipid contents (1441 cm^−1^, 2851 cm^−1^) were observed. In case of lung cancer cells treated for 48 hours^19^, changes at 783 cm^−1^, 830 cm^−1^, 1004 cm^−1^ and 1445 cm^−1^ were observed. Both of these results are similar to the presented outcome of this work. Moreover, the change of the nucleic acid content is conserved in the PCA loadings (Fig. 2b) and causes the shifts in the score plot (Fig. 2a). Concomitantly, the change of the cellular protein content due to higher DOX concentration is also noticeable in the spectral bands (Fig. 1b), as well as from the reconstructed spectra (Fig. 3b,d). There are potential explanations for the alterations in protein composition, e.g. protein signaling to the late stage of cell apoptosis, protein signaling to the cell apoptosis^16^ and anti-apoptotic protein generation resulting from DNA damage^17^. The change of the protein content is mainly reflected in the loading of PC1 (Fig. 2b), which substantially separates the viable cells from those of non-viable (Fig. 2a). The DOX concentrations also alter the cellular lipid contents (Fig. 3b,d). The change of lipid mainly occurs due to the formation of lipid vesicles in cells^6^, formation of lipid vesicles on the cell surface as well as blebbing of the cell membrane^8,18,19^. This change is mostly pronounced in non-viable cells in the sample population (Fig. 3b,d). Additionally, the contribution of the lipid content due to the treatment with increased DOX concentrations is more pronounced in the second loading (Fig. 2b). The percentages of viable cells in all samples were examined by cell viability tests, demonstrate a decrease of cell viability ratio with increasing amounts of DOX (Table 2). Based on a PCA–SVM model, which was trained with spectra from control samples and only one sample with 1 µM DOX of one biological replicate, it was possible to distinguish between the viable and the non-viable cells (Fig. 2c), ensuing percentages of cells belonging to each distinct class match well with values of the viability test reference (Table 2). Furthermore, both sets of spectra assigned by the chemometric model were analyzed separately to investigate the effect of DOX treatment concentrations (Fig. 3). With increasing DOX concentration, median scores of viable cell spectra move closer to the decision boundary – the boundary that determines the viability state of the cells (Fig. 3a). The observed shift is more pronounced regarding PC2 axis, which originates due to lipid and nucleic acid content. On the other hand, the non-viable cell spectra are closer to the decision boundary at low DOX concentrations (Fig. 3c). Corresponding score values spread significantly more along PC1 axis. Presented spectral changes are consistent with the findings of related studies in this field^5,26,30,33^.

## Conclusion

In this work we presented a newly developed HCA-RS platform, which allows using the full potential of Raman spectroscopy for the analysis of cells at different physiological conditions. The advantages of the approach are demonstrated in form of a label-free cell viability assay for the investigation of doxorubicin-induced effects in THP-1 cells. The developed platform allows performing an analytical series, i.e. a complete drug-concentration viability study, without any human intervention, and enables to sample a statistically significant number of cells at different physiological conditions in mixed populations. A PCA-SVM model was trained only with spectra from one biological replicate. Later, the model successfully estimated the percentage of cell viabilities in the mixed population of all other test conditions. Here, cell viability assessments using Raman spectral information show excellent agreement with a standard cell viability test. Furthermore, the effect of increasing DOX concentration was also evaluated for both of the cell stages - viable and non-viable cells. The spectral changes are correlated to the macromolecular changes, i.e. protein, lipid, nucleic acid content in cells with the increase of DOX concentration, which also indicates the DOX cellular interaction mechanism. Future development of the HCA-RS would include real-time analysis of cells – and push it further to the analysis of living cells, project the cell type assignment in the brightfield image instantly after the acquisition of the Raman spectra as well as type-based cell sorting using Raman spectral information. The capability of the newly developed HCA-RS platform to probe molecularly a large number of cells demonstrates the potential of Raman spectroscopy as an all-around tool for cell studies, eventually paving the way of Raman spectroscopy for biomedical and clinical applications one step further.

## Methods

### Cell preparation

THP-1 cells (ATCC® TIB202™), a monocytic cell line derived from acute leukemia cells, were sub-cultured every 2–3 days in RPMI 1640 GlutaMax™ (ThermoFisher Scientific)/10% FBS Superior (Biochrom, Merck)/Penicillin (100 U/ml)/Streptomycin (100 µg/ml) (Biochrom, Merck) at 37 °C/5% CO_2_. Cells (passage numbers 6–9) were then incubated in medium with different DOX concentrations. For this purpose, cell cultures were centrifuged, suspended in fresh medium and transferred to 25 cm^3^ cell culture flasks (Greiner Bio-One). For each experiment, DOX (Sigma Aldrich) concentrations were adjusted to 0 µM (2 negative control flasks), 0.05 µM, 0.1 µM, 0.5 µM and 1 µM in 5 ml cell suspension. After 48 h incubation cells of each flask were harvested by washing in 5 ml pre-warmed PBS and fixed in ice-cold Roti^®^*-* Histofix (Carl Roth) for 15 min. After washing and re-suspension in PBS, cells were ready for Raman spectroscopic analysis.

### Cell viability assessment

After 48 h incubation in different conditions aliquots were taken of each flask and 10 µl cell suspension stained with 10 µl Trypan Blue solution (0.4%) (ThermoFisher Scientific). Viable and non-viable cells were counted and pictures were taken using the Countess™ II Automated Cell Counter (ThermoFisher Scientific). The cell concentration of each sample before the DOX treatment was around 10^6^ cells/ml.

### Raman spectroscopy System

**Figure 5:**
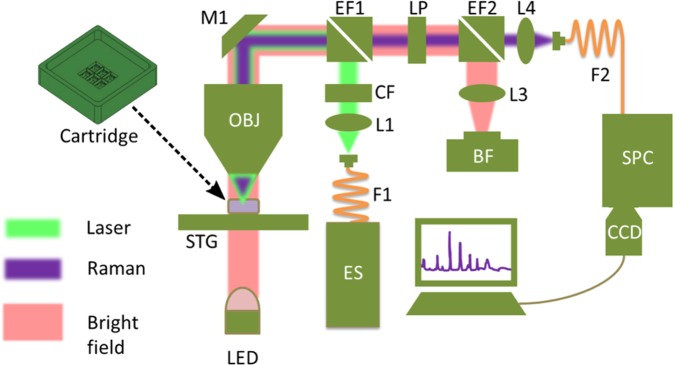
The experimental setup of HCA-RS system has many components. They are ES – excitation source, F – fiber, L – lens, CF – clean-up filter, EF – edge filter, M – mirror, OBJ – objective, STG – xyz stages, LP – long pass filter, BF – bright field camera, LED – light emitting diode, SPC – spectrograph and CCD – Raman spectral detector. The cartridge is placed on the STG and can accommodate six CaF_2_ coverslips for six different measurements in one run. The light green, the purple and the pink lines indicate the excitation, the Stokes Raman signal and the bright field illumination path, respectively. In this work we referred Stokes Raman as Raman signal which is generated in the sample plane and travels back with the excitation path up to EF1 and is then separated (along the light green line from the sample plane up to the EF1).

The experimental setup, shown in Fig. 5, consists of conventional Raman system building blocks, i.e. excitation source, pre-filter, optics for guiding light to the sample, collection optics, spectrograph and a detector. The excitation source ES (532 nm ± 0.5 nm fiber coupled laser DPSS series, LASOS, Germany), is coupled into the system via a multimode fiber F1 (62 µm, 0.22 NA FC-PC, Thorlabs, Germany). Afterwards, the laser beam is collimated by a lens L1 (focal length f = 25.4 mm, Thorlabs, Germany). Then a filter CF (532 nm ± 3.7 nm @ FWHM, SEMROCK, USA) is used to remove the unwanted spectral contributions from the in-coupling fiber. The collimated beam is guided to an objective lens OBJ (60x, 1 NA, water immersion, Nikon, Japan) via an edge filter EF1 (532 nm, SEMROCK, USA) and a mirror M1 (Thorlabs, Germany). The beam is expanded to a diameter of approximately 10 µm to illuminate the whole cell as proposed in^32,35^. In few situations, where the spectral acquisition from a small excitation volume reveals intracellular molecular heterogeneity, which could be significantly higher than the anticipated effects of an external disturbance, e.g. DOX-exposure. In^35^, different strategies to illuminate a large portion of the cell are outlined. The optical setup is built in such a way that the exit aperture of F1 and the sample plane are conjugated, meaning that the exit aperture of the fiber is imaged to the sample plane by means of the fiber-coupling lens and the objective lens. As a result, the 62 µm fiber is imaged to a 10 µm spot size. The expanded beam diameter enables acquisition of an average Raman signal from the cells in contrast to the Raman mapping, where the small laser spot scans the whole cell and later all the spectra are averaged for further analysis^35^. A red light emitting diode - LED (632 nm central wavelength, Thorlabs, Germany) is placed underneath a sample for the bright-field illumination – indicated by the pink line. After the interaction of the incoming laser with the sample, the both Rayleigh and Raman signal are generated and collected by the same objective lens OBJ. Both of the scattered signal from the sample are reflected back, guided by the mirror M1 and then they propagate through the edge filter EF1 (SEMROCK, USA) allowing only the Stokes Raman signal to pass, then through a low pass filter LP (SEMROCK, USA) and lastly another edge filter EF2 (SEMROCK, USA). EF2 guides the bright field illumination to a bright-field camera BF (Thorlabs, Germany) by a collimating lens L3 (f = 60 mm, Thorlabs, Germany). After passing through the EF1 and LP, the Raman signal lands on another focusing lens L4 (f = 30 mm, Thorlabs, Germany). L4 focuses the signal to a multimode fiber (105 µm, 0.1 NA, Thorlabs, Germany) guiding it to a spectrograph SPC (Spectrapro 2300i, Princeton Instruments, USA), which has a grating with 400 lines/mm. At the end of the spectrograph a cooled back-illuminated deep depletion charge-coupled device (CCD) (PIXIS100BR-DD, Princeton Instruments, USA) is placed to capture the diffracted Raman signal as an image. In addition, three motorized stages STG (two from Newport, USA and one from Thorlabs, Germany) are placed under the objective lens OBJ. They also hold the custom made sample holder – cartridge. The cartridge is compartmentalized for accomodation of different samples with different physiological properties, and the STG motors assist to place the sample under OBJ for the measurement. Two motorized stages are used to place the cells in the exact position under the laser excitation beam, and the third one places the sample in the proper focal plane for the optimal Raman signal acquisition. Moreover, the first two stages also assit movement from one sample type to the next sample type.

### Cartridge

A custom-made coverslip holder – cartridge (Fig. 5) - was designed considering the CaF_2_ coverslip dimensions and the limit of the x, y translational stages which can accommodate six 10 mm × 12 mm CaF_2_ coverslips (Sigma-Aldrich, Germany). It was made of acrylic (Poly methyl methacrycrylate - PMMA) to allow LED light transmission. The cartridge provides some space on the top and bottom of the Raman-grade CaF_2_ slides (S1 in supplementary document), which are filled with water to make sure that no air bubbles were present. Moreover, the provided spaces are sufficient enough to omit background contribution in the Raman signal from PMMA. The total dimension of the six wells are 32.5 mm × 27.6 mm, but the wells are positioned in such a way that the longest translation motion of the motorized stages falls within their limits, i.e. 25 mm from the first well to the last well. A boundary is also placed to the edge of the cartridge for containing water (the big boundary surrounding the whole cartridge in Fig. 5). To move from one sample to the next, the cartridge is moved down by the z motor, translated to the next sample by the xy motors and moved up by the z motor again. Without having water filled, the movements could provide the threat of mixing cells from one sample to other. Hence water filling removes the sample mixing threat keeping the OBJ in the water during entire measurement.

### Raman measurement

For the Raman measurement, Raman-grade CaF_2_ slides (Crystal, Germany) were used. At first, the coverslips are coated with poly-L-lysine to stabilize the cells as the rapid stage movements introduce wobbling of cells due to moment of inertia. Later the slides were placed in the cartridge and the cells were pipetted onto it. The top row of the cartridge contained a control, cells treated with 0.05 µM DOX and cells treated with 0.1 µM DOX. The wells of the bottom row contained cells treated with 0.5 µM DOX, cells treated with 1 µM DOX and another control. The spectral quality was ensured inspecting particular Raman band of the raw Raman spectra before performing the measurement. Moreover, the system estimated the proper focal plane to maximize the Raman spectral quality automatically. The measurement started at the top left well and ended at the bottom right well. The integration time and the laser power were set to 0.25 s and 96 mW at the end of the objective, respectively. For the DOX treated cells, 5 s of photo-bleaching time or waiting time was introduced. For each of the samples multiple settings of FOV, i.e. 10 × 10 frames and 5 × 5 frames were used so that approximately 1000 spectra were acquired from each of the cartridge sample wells. The whole measurement was performed for two different sets of biological replicates in multiple spatial locations for each of it.

### Data analysis

The data evaluation of Raman spectra has been previously reported^42^, and can be divided into two parts – spectral preprocessing and statistical analysis^43^. All data processing was performed in Matlab. The preprocessing includes wavelength calibration, intensity calibration, cosmic spike removal, background correction and normalization. The wavelength calibration relates the pixel number axis of the CCD-detector to wavenumber axis of the acquired Raman spectra. This was performed by assigning well-known band positions of paracetamol, following a polynomial interpolation. Intensity calibration was performed, using a NIST standard (National Institute of Standards and Technology, USA) white calibration lamp (Kaiser optical system, USA). NIST is more reliable and is based on the measurement of the luminescence of an intensity standard, whose relative irradiance has been determined. It provides a means to establish the instrument response function, which then serves to correct the sample spectrum to the true relative Raman intensity. In some cases, cosmic spikes were present in some spectra, and were removed by an in-house-developed cosmic spike removal algorithm. Afterwards, the background correction was performed using a third order extended multiplicative signal correction (EMSC) method^44^, taking into account the water spectrum, fluorescence contributions, and a pure cell spectrum. The water spectra were taken into consideration as the measurements were conducted in aqueous medium resulting background contribution from it in the acquired spectra. Afterwards, smoothing was implemented using Savitzky-Golay filter^45^ as well as area normalization. Area normalization normalized the acquired spectra considering the area under the background corrected spectra and diminishes the effect of excitation laser intensity fluctuation. A sample spectrum of 1 µM DOX treated THP-1 cell before and after the background correction are presented in Fig. S3 in supplementary document. To reduce the dimensionality of the data, principal component analysis (PCA) was used. For the classification of the viable and the non-viable cells in the mixed population, the control and the 1 µM DOX treated cells of batch 2 were used to train a SVM model, considering the first two components from PCA. Some of the parameters of the SVM training model were: a linear classifier, an alpha value of 0.5, usage of a solver following the iterative single data algorithm^39^. Afterwards a 10-fold cross validation was performed and later the model was applied to predict the percentage cell viability for both of the batches 1 and 2.

## Supplementary information

Supplementary Info

## Data Availability

The datasets generated during and/or analysis during the current study are available from the corresponding author on reasonable request.
