# Supplementary material for: New perspectives for viability studies with high-content analysis Raman spectroscopy (HCA-RS)
Source: Sci Rep. 2019 Sep 2;9:12653. doi: 10.1038/s41598-019-48895-7 (PMC6718400; doi:10.1038/s41598-019-48895-7)
Supplement: Supplementary file 1 — Supplementary Info [file 41598_2019_48895_MOESM1_ESM.pdf]

Supplementary Materials:

New perspectives for viability studies with high-content analysis Raman spectroscopy (HCA-RS)

Abdullah Saif Mondol[1,2], Natalie Töpfer[1,3], Jan Rüger[1,2], Ute Neugebauer[1,2,3], Jürgen Popp[1,2,3], and Iwan W. Schie[1]

1 Leibniz Institute of Photonic Technology and Leibniz Health Technologies, Albert Einstein Str. 9, 07745 Jena, Germany
2 Institute of Physical Chemistry and Abbe Center of Photonics, Friedrich-Schiller University Jena, Helmholtzweg 4, 07743 Jena, Germany
3 Center of Sepsis Control and Care, Jena University Hospital, Am Klinikum 1, 07747 Jena, Germany

Corresponding author: iwan.schie@leibniz-ipht.de

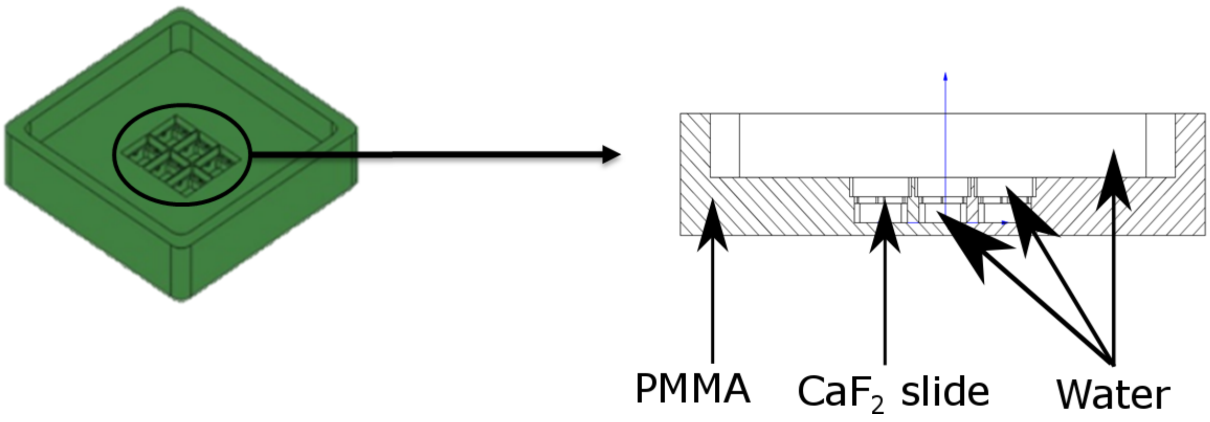

S1: The cross section of the cartridge shows the schematic of the design. It also indicates the position of the CaF_2 slide and the water.

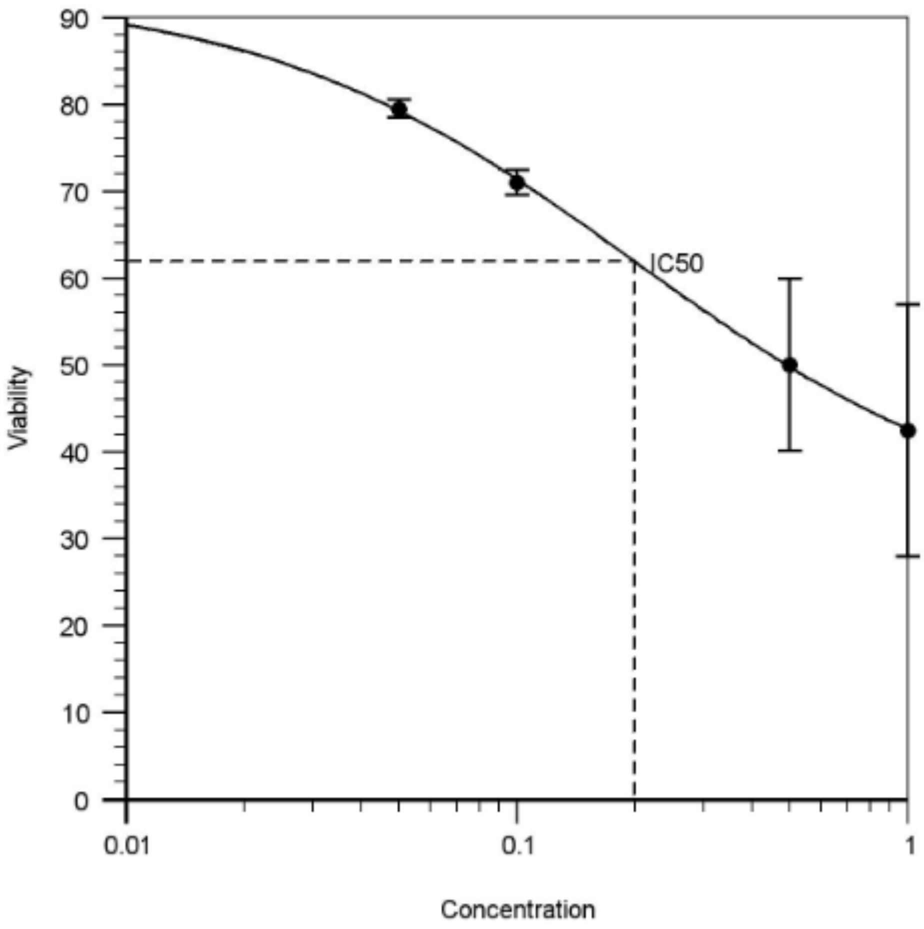

S: The cell viability vs. the DOX concentrations are plotted to calculate the IC_{50} value. The formula for the calculation is estimated from https://www.aatbio.com/tools/ic50-calculator

and represented as:

$$V = 30.918 + \frac{93.021 - 30.918}{1 + \left(\frac{Conc.}{0.199}\right)^{0.905}}$$

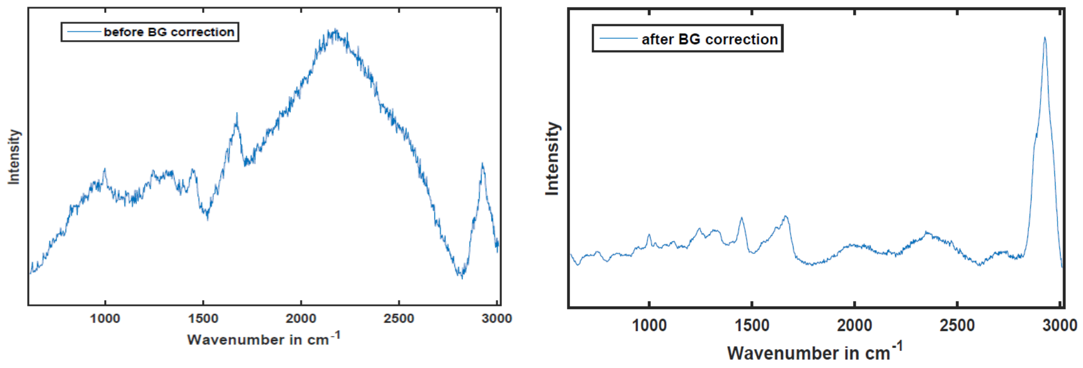

S: Sample acquired spectrum of a 1 µM DOX treated THP-1 cell before (left) and after (right) the background correction using EMSC.
